# Trends in Prescribing of Nicotine Replacement Therapy to Pregnant Women in Primary Care in England

**DOI:** 10.1093/ntr/ntab037

**Published:** 2021-03-05

**Authors:** Lisa Szatkowski, Luis Reeves Vaz, Linda Fiaschi, Laila Tata, Tim Coleman

**Affiliations:** 1 Division of Epidemiology and Public Health, School of Medicine, University of Nottingham, Nottingham City Hospital, Clinical Sciences Building, Nottingham, UK; 2 Division of Primary Care, School of Medicine, University of Nottingham, Medical School, Queen’s Medical Centre, Nottingham, UK; 3 Leicester Real World Evidence Unit, University of Leicester, Leicester General Hospital, Leicester, UK; 4 Nottingham Clinical Trials Unit, University of Nottingham, University Park, Nottingham, UK

## Abstract

**Introduction:**

Smoking during pregnancy remains common, and the English National Health Service (NHS) has recently been directed to prioritize providing cessation support for pregnant women. We investigated the impact on prescribing of stop smoking treatments to pregnant women of the 2013 transfer of public health budgets from the NHS to administrative authorities responsible for local social care and other nonhealth services (local authorities).

**Methods:**

We used data from the Clinical Practice Research Datalink and Hospital Episode Statistics to determine annual proportions (2005–2017) of women who smoked during pregnancy and who were prescribed, at least once before childbirth, (1) any NRT and (2) long- and short-acting NRT together (dual NRT). Segmented regression was used to quantify the impact of the 2013 transfer of smoking cessation budgets to local authorities, assessing changes in the level and the trend of the proportions post-2013 compared with pre-2013.

**Results:**

We identified 84 539 pregnancies in which women were recorded as smoking; any NRT was prescribed in 7.9% (*n* = 6704) and dual NRT in 1.7% (*n* = 1466). Prescribing of any NRT was declining prior to 2013 at an absolute decrease of −0.25% per year, but the rate of decline significantly increased from 2013 onwards to −1.37% per year. Prescribing of dual NRT was increasing prior to 2013 but also decreased post-2013.

**Conclusions:**

These findings suggest that transferring responsibility for English Smoking Cessation Services from the NHS to local authorities adversely affected provision of cessation support in pregnancy. Consequently, some women may have been denied access to effective cessation treatments.

**Implications:**

Women who smoke during pregnancy may be being denied potentially effective means to help them quit, contrary to NICE guidance, at what can be a teachable moment with substantial immediate and longer-term health benefits for woman and their unborn child, and economic benefits for the NHS. When the organizations responsible for offering smoking cessation support are changed, health systems should consider potential adverse effects on the delivery of support and deploy strategies for mitigating these.

## Introduction

Globally, smoking in pregnancy remains common.^1^ In England in the year to March 2020, 10.4% of women smoked at the time of delivery^2^ and in 2015, 23.3% of UK women were estimated to have smoked (any frequency/quantity) in pregnancy.^1^ Smoking in pregnancy is associated with increased risks of many adverse pregnancy, birth, and child outcomes^3^ and children of smoking mothers are twice as likely to themselves start smoking.^4^ Reducing smoking in pregnancy is likely to have a major impact on medical resource use and the English NHS has recently been directed to prioritize providing cessation support for pregnant women.^5^

Nicotine replacement therapy (NRT) can assist smoking cessation in pregnancy,^6^ though due to insufficient data on safety, its use is suggested where smoking cessation without NRT fails.^7(p26)^ Adherence with NRT in pregnancy is poor, potentially because nicotine metabolism is faster in pregnancy and standard doses of NRT are thus less likely to ameliorate withdrawal symptoms.^8^ Dual NRT, combining a nicotine patch (for a steady background nicotine supply) with a fast-acting NRT like gum (to “top-up” the nicotine dose as needed), may be more likely to lead to cessation than mono NRT,^9^ though current NHS guidelines for supporting smoking cessation in pregnancy do not mention use of dual NRT as a treatment option.^7(p26)^

A recent British Lung Foundation report suggests that, since 2013 when public health budgets were transferred from the NHS to local authorities, primary care prescribing of stop smoking treatments to people who smoke may have declined.^10^ This report used data on items dispensed and estimates of population size and smoking prevalence and did not formally quantify the impact of funding changes using robust statistical methods. Hence, here we use interrupted time series analysis with data routinely recorded in primary care to investigate trends in primary care prescribing of NRT to pregnant women before and after 2013.

## Methods

We used data from all (*n* = 398) general practices in England who contribute data to the Clinical Practice Research Datalink (CPRD)^11^ and which are linked to secondary care data from Hospital Episode Statistics (HES),^12^ allowing us to identify mothers and their children. Women were included in the study cohort if they had one or more pregnancies resulting in a live birth or stillbirth recorded in CPRD-HES between January 1, 2005 and December 31, 2017 and if they were aged 15–49 at the time of birth. Women were identified as smoking in pregnancy if they had a diagnostic code indicating current smoking, or a prescription for a smoking cessation medication, recorded at least once during gestation. Prescriptions for NRT were identified using relevant Multilex drug codes. Dual NRT was defined as prescription of a long-acting transdermal nicotine patch and a short-acting formulation (eg, gum, lozenge, inhalator, tablet, or spray) on the same day.

By year of delivery, we determined annual proportions of women who smoked during pregnancy who were prescribed (1) any NRT at least once during pregnancy and (2) dual NRT at least once during pregnancy; by definition, these groups were not discrete—women who were prescribed dual NRT were a subset of those prescribed any NRT. We used segmented regression^13^ to quantify the impact on each data series of the 2013 transfer of smoking cessation budgets to local authorities, assessing changes in the level and the trend of the data post-2013 compared with pre-2013. We used a backward elimination approach to build parsimonious models and checked for autocorrelation by visual inspection of the autocorrelation function of the model residuals and by conducting a Portmanteau test using a significance level of *p* < .05.

Data management and analyses were conducted using Stata 16 (Stata Corp, College Station, TX). CPRD-HES data received ethical approval from a National Research Ethics Service Committee (NRES) for data collection and subsequent observational research using anonymized data; individual studies do not require further separate ethical approval. The protocol for this project was approved by the CPRD Independent Scientific Advisory Committee (reference: 16_151).

## Results

**Figure 1.**
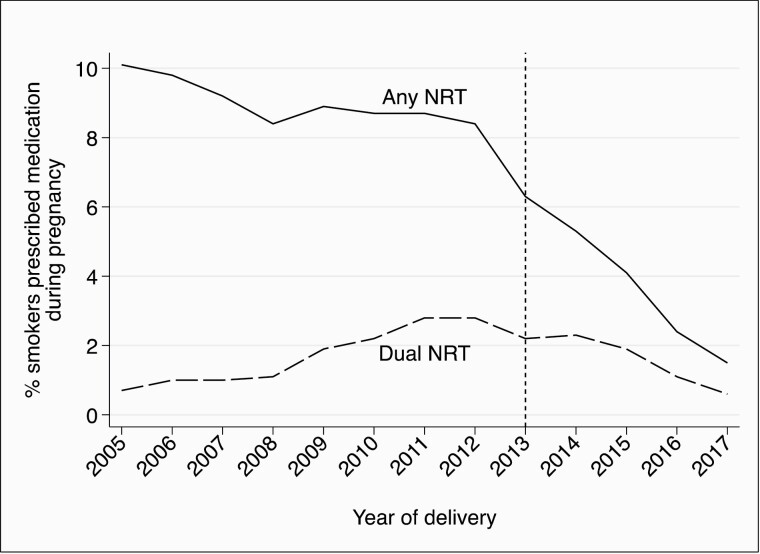
Prevalence of prescribing of any nicotine replacement therapy (NRT) and dual NRT to pregnant smokers by year of delivery (dashed vertical line represents transfer of smoking cessation budget to local authorities).

We identified 339 875 pregnancies during the study period, and in 84 539 (24.9%), the mother was recorded as smoking; this prevalence was stable at an average 25.6% for the first 5 years of the study period before declining to 21.3% in 2017. Overall, any NRT was prescribed in 7.9% (*n* = 6704) and dual NRT in 1.7% (*n* = 1466), of pregnancies where the mother smoked. Figure 1 shows changes over time in the percentage of pregnancies where the mother smoked where any NRT and dual NRT were prescribed.

Table 1 presents results from the interrupted time series analysis showing the year-on-year absolute change in the prevalence of prescribing of any NRT and dual NRT between 2005 and 2012, the absolute change in trend from 2012 to 2013, and the year-on-year absolute change in prescribing from 2013 onwards.

**Table 1.** Year-on-Year Percentage Changes in Prescribing Before and After the 2013 Transfer of Smoking Cessation Budgets to Local Authorities

Medication	Absolute annual percentage change in prescribing 2005–2012 (95% CI)	Change in trend from 2012 to 2013 % (95% CI)	Absolute annual percentage change in prescribing 2013–2017^a^ (95% CI)
Any NRT	−0.25 (−0.36 to −0.15), *p* < .001	−1.12 (−1.35 to −0.88), *p* < .001	−1.37 (−1.52 to −1.21), *p* < .001
Dual NRT	0.34 (0.26 to 0.42), *p* < .001	−0.76 (−0.93 to −0.60), *p* < .001	−0.42 (−0.53 to −0.31), *p* < .001

CI, confidence interval; NRT, nicotine replacement therapy.

^a^Annual change 2013–2017 = annual change 2005–2012 + change in trend 2012–2013.

Findings show that prescribing any NRT to pregnant women who smoked was declining prior to 2013 at a rate of approximately one quarter of a percentage point per year. However, the rate of decline increased from 2013 onwards such that from 2013 to 2017 prescribing decreased by an absolute magnitude of 1.37% per year. Prescribing of dual NRT was increasing prior to 2013, but there was a similar change in trend such that from 2013 to 2017 prescribing decreased by an absolute magnitude of 0.42% per year.

## Discussion

Using data from a large, representative data set, we have shown a decline in primary care prescribing of any NRT to pregnant women who smoke, with the rate of decline increasing since 2013. Prescribing of dual NRT, potentially a more effective means of cessation support than mono NRT, was increasing up to 2012, but this too has since decreased. This is despite smoking in pregnancy remaining a significant problem; within our data set, at the end of the study period, approximately one in five women smoked during pregnancy.

Though we cannot assume causation, the timing of declines in prescribing suggests an association with the 2013 funding changes. It is possible that some of the decline in prescribing of NRT is related to increased use of electronic cigarettes; the number of e-cigarette users in the general population in Great Britain increased from 0.7 million in 2012 to 2.9 million in 2017.^14^ However, in the UK, use of e-cigarettes by pregnant women is not particularly widespread; a 2017 survey found that only 5% of pregnant women reported any vaping during pregnancy and only around 1% reported exclusive vaping (ie, they had stopped smoking).^14^ It seems unlikely, therefore, that vaping behavior would have had a substantial impact on pregnant women’s propensity to use NRT and hence, on primary care prescribing. Nevertheless, research investigating any relationships between smoking, e-cigarette, and NRT use by pregnant women, including impacts of longitudinal changes in prevalence of use and potential trade-offs between e-cigarette use and primary care NRT prescribing, would be informative.

It is also possible that some of the decline in prescribing may have been offset by increased referral of smokers to the post-2013 local authority-commissioned Stop Smoking Services (SSS). We did not have data available on direct referrals from primary care to SSS. However, data on the number of people setting a quit date in SSS based in primary care, community and pharmacy settings (which together comprise over 90% of all quit dates set with SSS) show a steady decline from the 2011/2012 financial year to 2016/2017. For example, 332 011 people set a quit date in primary care-based SSS in 2011/2012 which fell to 115 460 in 2016/2017; declines were of a similar magnitude in community-based and pharmacy-based SSS.^15^ These figures suggest there was an overall decline in the use of SSS and do not provide any support for the notion that referrals from primary care increased.

Around 20% of SSS provide NRT to pregnant women via FP10 prescriptions issued through primary care,^16^ and this figure has not changed substantially over time (R Thomson, personal communication, February 3, 2021). The number of pregnant women who set a quit date with SSS fell from 26 080 in 2011/2012 to 15 216 in 2016/2017,^15^ and we estimate that this would result in approximately 2000 (ie, 26 080 − 15 216 × 0.2) fewer women annually being prescribed NRT through SSS via FP10 prescription. This is substantially less than the overall decline in prescribing of NRT in primary care and suggests that there must also be a decline in direct prescribing of NRT by general practitioners, outwith any prescribing through SSS. Since 2013, many local authority-commissioned SSS have been cut or decommissioned.^10,17^ It is possible that where there is no local SSS to which general practitioners can refer pregnant women there is a reduced stimulus for discussion of smoking cessation and thus less direct prescribing of NRT. This may indicate an indirect effect of decommissioning SSS on smoking cessation activity and hence on NRT prescribing in primary care too.

Given the lack of evidence to support alternative explanations for the reduction in primary care NRT prescribing to pregnant women, it seems most likely that this is related to the transfer of public health budgets to local authorities. There is little available data on how the specific provision of LA SSS support to pregnant women might have changed in this period. Anecdotal reports suggest that some local authorities have prioritized providing SSS support to pregnant women who smoke, as well as to other disadvantaged groups. However, it seems likely that there has been a parallel decrease in SSS support for pregnant smokers and this has been reflected in reduced primary care prescribing.

Therefore, contrary to NICE guidance, some pregnant women, unable to access NRT through either primary care or a specialist SSS, may have been denied access to effective cessation support.^7^ Future NHS provision of such support, advocated in the NHS Long Term Plan, may improve this situation.^5^ However, maximal immediate and longer-term health benefits for women and their unborn children, and economic benefits for the NHS, are unlikely to follow this investment unless the decline in local authority provision of stop smoking support for pregnant women is reversed.

## Supplementary Material

A Contributorship Form detailing each author’s specific involvement with this content, as well as any supplementary data, are available online at https://academic.oup.com/ntr.

ntab037_suppl_Supplementary_Taxonomy_Form

## References

[1] Lange S, Probst C, Rehm J, Popova S. National, regional, and global prevalence of smoking during pregnancy in the general population: a systematic review and meta-analysis. Lancet Glob Health. 2018;6(7):e769–e776. 29859815 10.1016/S2214-109X(18)30223-7

[2] NHS Digital. Statistics on Women’s Smoking Status atTime of Delivery: England Quarter 4, 2019–20. NHS Health and Social Care Information Centre. 2020. https://digital.nhs.uk/data-and-information/publications/statistical/statistics-on-women-s-smoking-status-at-time-of-delivery-england. Accessed November 26, 2020.

[3] Royal College of Physicians. Hiding in Plain Sight: Treating Tobacco Dependency in the NHS. 2018. https://www.rcplondon.ac.uk/projects/outputs/hiding-plain-sight-treating-tobacco-dependency-nhs. Accessed November 26, 2020.

[4] Leonardi-Bee J, Jere ML, Britton J. Exposure to parental and sibling smoking and the risk of smoking uptake in childhood and adolescence: a systematic review and meta-analysis. Thorax. 2011;66(10):847–855. 21325144 10.1136/thx.2010.153379

[5] NHS England. The NHS LongTerm Plan. 2019. https://www.longtermplan.nhs.uk/. Accessed November 26, 2020.

[6] Claire R, Chamberlain C, Davey MA, et al. Pharmacological interventions for promoting smoking cessation during pregnancy. Cochrane Database Syst Rev. 2020. doi:10.1002/14651858.CD010078.pub3 PMC7059898 32129504

[7] National Institute for Health and Care Excellence. Smoking: Stopping in Pregnancy and after Childbirth. Public Health Guideline PH26. 2010. https://www.nice.org.uk/guidance/ph26. Accessed November 26, 2020.

[8] Bowker K, Lewis S, Coleman T, Cooper S. Changes in the rate of nicotine metabolism across pregnancy: a longitudinal study. Addiction. 2015;110(11):1827–1832. 26119134 10.1111/add.13029 PMC5014174

[9] Brose LS, McEwen A, West R. Association between nicotine replacement therapy use in pregnancy and smoking cessation. Drug Alcohol Depend. 2013;132(3):660–664. 23680076 10.1016/j.drugalcdep.2013.04.017

[10] British Lung Foundation. Less Help to Quit: What’s Happening to Stop Smoking Prescriptions Across Britain. 2018. www.blf.org.uk/policy/less-help-to-quit. Accessed November 26, 2020.

[11] CPRD. Clinical Practice Research Datalink. https://cprd.com/home. Accessed November 26, 2020.

[12] Health and Social Care Information Centre. Hospital Episode Statistics. http://www.hscic.gov.uk/hes. Accessed November 26, 2020.

[13] Wagner AK, Soumerai SB, Zhang F, Ross-Degnan D. Segmented regression analysis of interrupted time series studies in medication use research. J Clin Pharm Ther. 2002;27(4):299–309. 12174032 10.1046/j.1365-2710.2002.00430.x

[14] Bowker K, Lewis S, Phillips L, et al. Pregnant women’s use of e-cigarettes in the UK: A cross-sectional survey. BJOG Int J Obstet Gynaecol. 2020. doi:10.1111/1471-0528.16553 33012050

[15] NHS Digital. Statistics on NHS Stop Smoking Services in England. https://digital.nhs.uk/data-and-information/publications/statistical/statistics-on-nhs-stop-smoking-services-in-england. Accessed February 4, 2021.

[16] Fahy SJ, Cooper S, Coleman T, Naughton F, Bauld L. Provision of smoking cessation support for pregnant women in England: results from an online survey of NHS Stop Smoking Services for Pregnant Women. BMC Health Serv Res. 2014;14:107. 24593130 10.1186/1472-6963-14-107 PMC3975862

[17] Cancer Research UK, Action on Smoking and Health. Feeling the Heat: The Decline of Stop Smoking Services in England; 2018. https://www.cancerresearchuk.org/sites/default/files/la_survey_report_2017.pdf. Accessed November 26, 2020.

